# Anesthetic management in a patient with severe tracheal stenosis by monitoring oxygen reserve index

**DOI:** 10.1186/s40981-022-00562-z

**Published:** 2022-09-15

**Authors:** Sho Matsuba, Mitsuki Sawai, Saki Higashitani, Fumiya Sawasaki, Hiromasa Kida, Kan Takahashi

**Affiliations:** Department of Anesthesiology, Kanazawa Medical University, 1-1, Daigaku, Uchinada-cho, Kahoku-gun Ishikawa 920-0293 Japan

**Keywords:** Tracheal stent placement, General anesthesia, Respiratory management, ORi™, IPV®

## Abstract

**Background:**

General anesthesia for tracheal stenting is challenging because of difficult ventilation and accompanying hypoxia. We report the use of oxygen reserve index (ORi™) during tracheal stenting.

**Case presentation:**

Cauterization of an intratracheal tumor and tracheal stenting was scheduled in a patient. ORi decreased from 0.3 to 0.2 after starting cauterization using a flexible bronchoscope through a tracheal tube with 28% oxygen, while SpO_2_ was maintained at 100%. ORi further decreased to 0, followed by a decrease of *SpO*_*2*_ < 90%, and surgery was interrupted. SpO_2_ was increased shortly after increasing FiO_2_ to 1.0, but ORi remained 0 when surgery was resumed; it was increased after completion of cauterization. Both ORi and SpO_2_ were maintained above 0.4 and 98%, respectively, during tracheal stenting through a rigid bronchoscope under intrapulmonary percussive ventilation.

**Conclusion:**

ORi was useful for predicting a decrease of SpO_2_ under general anesthesia for tracheal stenting.

## Background

Tracheal stenting is used to maintain airway in cases of stenosis due to tumor; however, anesthesia management for tracheal stent placement has not been established. Respiratory management for adverse events, such as ventilatory insufficiency and hypoxemia, is a problem during the surgery [1]. Anesthetic management with preserving spontaneous breathing or with controlled ventilation is controversial [2, 3]. Early detection of the hypoxic critical point to interrupt the surgical procedure and perform urgent treatment is required for anesthesia management in this surgery.

## Case presentation

A 70-year-old man was diagnosed with recurrence of right upper lobe lung cancer. Four years ago, he underwent thoracoscopic right upper lobectomy and lymph node dissection for squamous cell carcinoma of the right upper lobe. He visited the hospital with complaints of dyspnea and bloody sputum. Bronchoscopy showed compression of the carina by a hemorrhagic tumor. Tracheal stenting to improve respiratory distress was scheduled under general anesthesia with monitoring of oxygen reserve index (ORi™) (Masimo Corp., Irvine, CA, USA) for indexing respiratory condition and possible hypoxia. ORi™ was measured noninvasively and continuously with a sensor on the left second finger by the pulse oximetry (Radical-7™, Masimo Corp; Irvine, CA, USA). SpO_2_ was at 96–98% in the room air, but he had stridor on auscultation and orthopnea.

Before induction of general anesthesia, catheters were placed into bilateral femoral veins under local anesthesia in preparation for extracorporeal membrane oxygenation during surgery. An arterial pressure line was inserted into the left radial artery. General anesthesia was induced using 50 μg of fentanyl, 3 μg/ml (TCI) of propofol, and 50 mg rocuronium. Airway was secured by intubating a spiral tube (diameter 9 mm, Parker Medical, USA). Anesthesia was maintained using propofol administration at 2.1–3 μg/ml (TCI) and remifentanil, and the depth of anesthesia was adjusted with reference to the bispectral index (BIS).

**Fig. 1:**
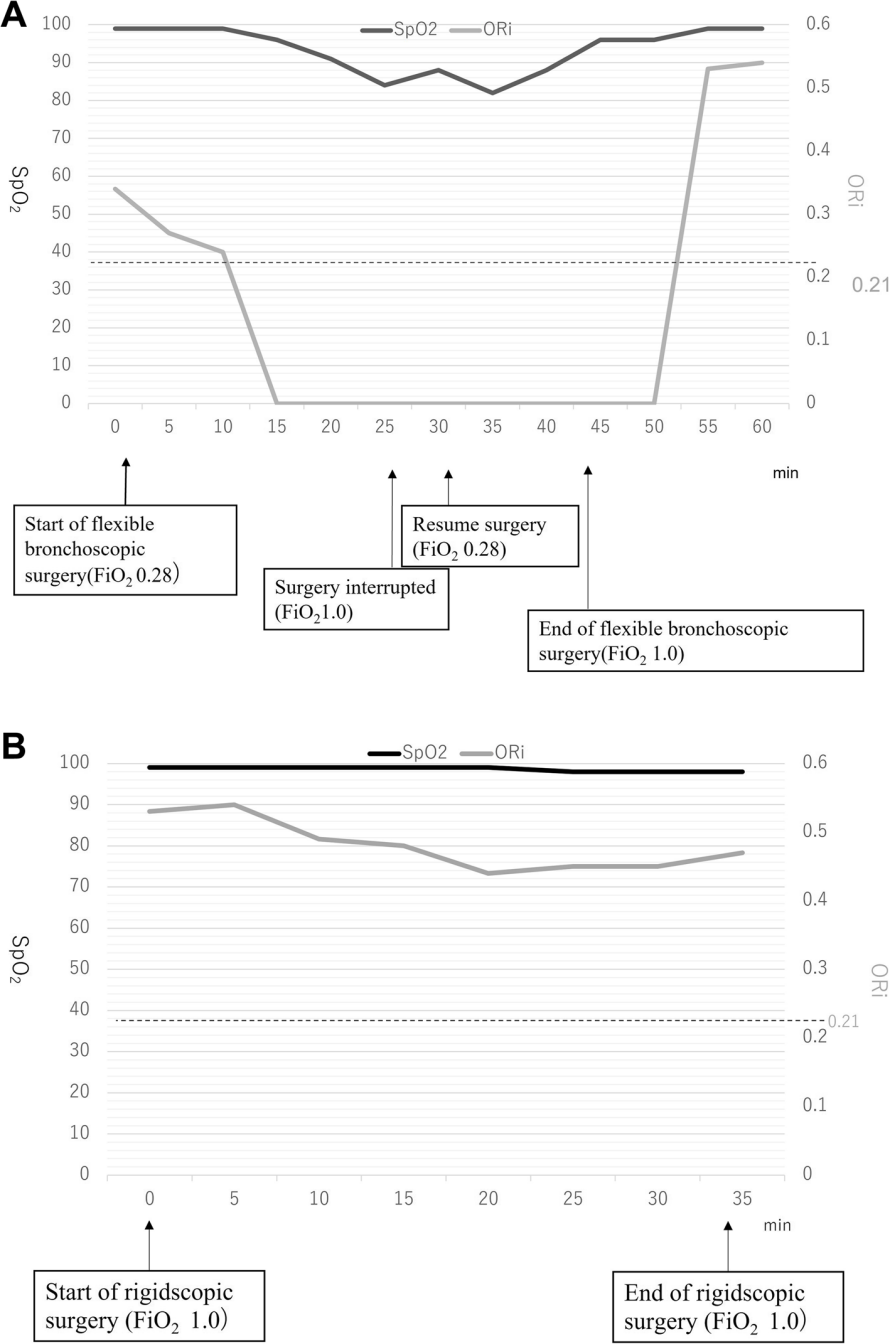
Oxygen reserve index (ORi™) and peripheral oxygen saturation during surgery using flexible (**a**) and rigid (**b**) bronchoscopes. **a** ORi reduced immediately after starting surgery. It decreased to 0.21, when SpO2 began to decrease. There were few minutes between the onset of decrease of ORi and of SpO2. **b** ORi was maintained above 0.4, and SpO2 did not decrease during surgery with a rigid scope

First, a flexible bronchoscope was inserted via an adaptor (Bodai Y connector®, Independence Australia Group, Australia) through the tracheal tube to cauterize and reduce the tumor size. As argon plasma coagulation was used, FiO2 was lowered to 0.28 for preventing airway fire. However, due to the large size of the bronchoscope relative to the inner diameter of the tracheal tube, tidal volume was significantly reduced. ORi decreased from 0.3 to 0.2 within few minutes after starting surgery (Fig. 1a). SpO2 started to decrease when ORi was 0.2; however, it was > 90%; even ORi further decreased to 0. SpO2 decreased < 90% approximately 5 min later, when surgery was interrupted. After confirming that SpO_2_ recovered by raising FiO_2_ to 1.0, the surgical procedure for tumor resection was resumed. However, due to bleeding from the tumor, there was not enough time for oxygenation to recover SpO_2_ at 100%. Therefore, when SpO_2_ recovered slightly, FiO_2_ was lowered to 0.28 again, and surgery was resumed. After the procedure was completed, FiO_2_ was raised to 1.0. Then, SpO_2_ in 5 min recovered to 99–100%, followed by an increase in ORi. About 5 min after SpO_2_ reached 100%, ORi rose to 0.53 (Fig. 1a).

Next, the flexible scope was extubated and replaced with a rigid scope, and a tracheal stent was placed using the rigid scope. When using a rigid scope, the ventilation volume may decrease, so we used an intrapulmonary percussive ventilator (IPV®, MODEL IPV-1, Percussionaire Corp; Sagle, ID, USA). A breathing circuit was connected directly to the rigid scope, and an IPV was connected to the inspiratory side of the circuit for percussion ventilation. At first, ventilation was performed only pressure-controlled ventilation (PCV:Pi 14 cm H2O, RR15/min, *IE* = 1:2, PEEP 5 cm H2O), but since the tidal volume decreased to 90 ml, IPV (operating pressure 30 psi, percussion frequency 3 Hz. The optimal drive pressure for IPV is 35–40 psi for adults, but high pressure could interfere with surgical procedure, so we started at 30 psi) was immediately started. FiO_2_ was managed at 1.0 during rigid bronchoscopic surgery as there is no longer a possibility of airway fire. Applying IPV enabled the maintenance of sufficient oxygenation during rigid bronchoscopy. The level of ORi did not decline, and SpO_2_ remained above 98% (Fig. 1b). The surgery was completed with a tracheal stent that was placed without complications. Awakening was good, and symptoms of respiratory distress improved immediately after the operation. The patient woke up from general anesthesia without any symptoms of respiratory distress.

## Discussion

Intratracheal surgery has become more common in recent years for patients with airway narrowing diseases. When radical surgery with tracheoplasty/bronchoplasty is impracticable because of tumor progression, tracheal stent placement may be performed as an emergency measure; however, there is no established method of anesthesia for the procedure. Inadequate ventilation and hypoxia are often the most critical problems in anesthesia management. Previous studies reported that both spontaneous breathing under local anesthesia and controlled ventilation under general anesthesia could be safely carried out [4–7]. In our patient, we considered that the tumor was hemorrhagic, and that body movement would interfere with the surgical operation, so we chose to manage the patient by general anesthesia with controlled ventilation using a muscle relaxant. When evaluating hypoventilation/non-ventilation associated with surgical operation using SpO_2_ as an index, the time from when SpO_2_begins to decrease to when it falls into hypoxemia is short, and there is a possibility that the response will be delayed [8]. Thus, we used ORi™ for monitoring in this surgery [9, 10].

ORi™ is a noninvasive parameter to predict PaO_2_ between 100 and 200 mmHg, and it is possible to continuously monitor an oxygen state of SpO_2_ 98% or more, and it can also be used as a predictor of SpO_2_ decrease. In a state of SpO_2_ 98% or more, if ORi™ is 0.24 or more, it represents PaO_2_ 100 mmHg or more, and if ORi™ is 0.55 or more, it represents PaO_2_ 150 mmHg or more; moreover, it was reported that up to PaO_2_240 mmHg showed a positive correlation function [11]. It is possible to take early measures to avoid hypoxemia by taking advantage of the characteristic that ORi™ begins to decrease before SpO_2_begins to decrease [12, 13]. Koishi et al. reported that ORi™ was useful to detect decrease in PaO_2_ much earlier than SpO_2_during one-lung ventilation [14]. They suggested that the use of ORi™ may reduce the risk of complication during OLV. Therefore, we also used ORi™ in our patient to predict severe hypoxia earlier than only using SpO2, which was used as an alarm for suspension of procedure to recover oxygenation. During flexible scope procedure, when ORi™ decreased to 0.21, SpO_2_ began to decrease from 100%. Although SpO_2_ remained at 90% after ORi™ showed 0, it dropped to less than 90% after 5 min. We estimated that the patient could tolerate the procedure, as 5 min later, ORi™ was 0.21. In this case, SpO_2_ decreased during flexible scope procedure, even though ORi was used to predict SpO_2_ decrease. The anesthesiologist predicted hypoxemia by the ORi and informed the surgeon, but the SpO2 remained high when the ORi began to decline, so the surgeon decided to continue the operation. When ORi began to fall, it was just when the tumor was being laser cauterized, so it was not possible to interrupt the surgery immediately. Through this case, we learned the importance of communication between anesthesiologists and surgeons during surgery.

Since we understood the time from ORi decrease to SpO_2_ decrease in this patient, we were able to manage anesthesia without causing more severe hypoxemia by requesting the operation to be stopped earlier.

IPV is an artificial respiration method that simultaneously performs intrapulmonary percussion therapy, high-frequency positive pressure ventilation, and aerosol inhalation. Its range of application is wide, and the percussion flow can promote oxygenation and carbon dioxide gas discharge in the alveoli [15]. IPV provides high-frequency breathing of 60–600 cycles/minute, thereby facilitating the elimination of airway secretions, and positive pressure by IPV provides more uniform ventilation in the alveoli and may improve gas exchange [16]. IPV is often used in surgery that causes poor ventilation, such as bronchial formation and tracheal stenting; however, there have been few reports of using IPV while measuring ORi. Based on the changes in ORi and SpO_2_ during flexible scope procedure, we monitored oxygenation during rigid scope procedure. We prepared to suspend the procedure if the level of ORi indicated 0.21 to avoid critical hypoxia. SpO_2_level was maintained at almost 100%, and ORi level did not decrease by more than 0.4 during rigid scope procedure, which suggests that IPV effectively preserved oxygenation. IPV was started at a lower pressure to prevent lung hyperinflation and obstruction of the surgical procedure, but oxygenation was adequately maintained, and no further pressure was needed. The reason for using IPV and PCV together was that we expected that PCV could maintain high airway pressure and prevent alveolar collapse. IPV is considered to have the effect of keeping the mean airway pressure constant at the alveolar level, and this case suggests that it has the effect of maintaining oxygen reserve [17].

## Conclusion

Tracheal stent placement could be safely managed with general anesthesia. IPV was useful to maintain oxygenation in rigid bronchoscopic procedure, and ORi was useful for early detection of hypoxia throughout tracheal stenting.

## Data Availability

Please contact the author for data requests.
